# Factors associated with perceived fear of future pandemics and/or epidemics: a cross-sectional study in Cyprus

**DOI:** 10.1038/s41598-023-39381-2

**Published:** 2023-07-27

**Authors:** Romina Alexandrou, Maria Kyprianidou, Galatia Photiou, Angelos P. Kassianos, Konstantinos Giannakou

**Affiliations:** 1 Department of Health Sciences, School of Sciences, European University Cyprus, Nicosia, Cyprus; 2 Cyprus International Institute for Environmental and Public Health, School of Health Sciences, Cyprus University of Technology, Limassol, Cyprus; 3 Department of Nursing, School of Health Sciences, Cyprus University of Technology, Limassol, Cyprus

**Keywords:** Epidemiology, Health policy, Public health, Human behaviour

## Abstract

This study aims to understand the levels of fear experienced by individuals regarding future pandemics and/or epidemics among the general population of Cyprus and comprehensively examine the diverse factors that influence this perceived fear. The cross-sectional study was conducted from October 1st, 2022, to February 19th, 2023. A proportionate quota sampling method was used for the recruitment, by recruiting a fixed number of participants from each age group, sex, and place of residence. The study collected information on sociodemographic and health-related characteristics, health literacy, trust, COVID-19 vaccination information, and perceived fear of future epidemics and/or pandemics using a self-administered questionnaire. The survey included 1075 participants, with 53.7% of them reporting fear of future pandemics. Logistic regression analysis revealed that women (OR = 2.37, 95% CI 1.78, 3.16) and individuals vaccinated against COVID-19 (OR = 1.57, 95% CI 1.02, 2.43) were significantly more likely to experience fear of future pandemics. Moreover, higher levels of trust (OR = 1.04, 95% CI 1.02, 1.06) and higher health literacy (OR = 1.05, 95% CI 1.03, 1.08) were associated with an increased likelihood of fearing future pandemics. Conversely, unemployment (OR = 0.30, 95% CI 0.13, 0.65) and having a postgraduate education decreased the likelihood of fearing future pandemics (OR = 0.56, 95% CI 0.34, 0.90). The linear regression model revealed that older age (β = − 0.10, 95% CI − 0.14, − 0.05) was negatively associated with a higher score of fear regarding future pandemics. Conversely, being in a vulnerable group (β = 2.02, 95% CI 0.75, 3.28) and having at least one chronic disease (β = 1.76, 95% CI 0.68, 2.84) showed positive associations with increased fear of future epidemics and/or pandemics. The findings emphasize the need for relevant authorities to prioritize mental health and disseminate information in a manner that avoids spreading fear and panic, particularly among vulnerable population groups.

## Introduction

Undoubtedly, one of the biggest global challenges in infectious disease management is dealing with the severe acute respiratory syndrome coronavirus 2 (SARS-CoV-2), which causes the disease COVID-19^1^. The first cases appeared in China in late 2019 and quickly spread throughout the world^2^. While anyone can contract COVID-19, individuals with underlying health conditions or the elderly are at higher risk of experiencing complications and even death^3^.

The COVID-19 pandemic has had far-reaching consequences beyond infections and deaths. The virus’s rapid spread in just a few weeks had a significant impact on nations worldwide, particularly in their ability to manage healthcare demands^4^. The pandemic also highlighted deficiencies in global medical care infrastructures, emphasizing the need to reorganize medical systems to better manage a pandemic while still providing general and specialized medical care^5^. Effective control of new infectious disease outbreaks will require redesigned public healthcare systems that prioritize better management, information, protection, support, and containment^6^.

The COVID-19 pandemic control measures implemented worldwide to halt the spread of virus have had profound and far-reaching negative impacts across economic, social, political, and psychological domains^7^. Research conducted in Italy and the UK has revealed that lockdown measures exacerbated depressive symptoms, sleep conditions, quality of life, and anxiety levels^8,9^. In contrast, a study conducted in Cyprus during the COVID-19 lockdown reported low levels of perceived stress, while research in Vietnam found decreased levels of depression, anxiety, and worry during partial lockdown^10,11^. Furthermore, social distancing measures have been associated with adverse effects on mental health, including depression, social anxiety, and psychosis^12,13^. However, mask use has shown potential positive effects on mental health during the pandemic^14^. Moreover, the global economic crisis caused by the pandemic has triggered significant shocks throughout the world economy, exacerbating inequalities within and between nations^15^. The combination of increased social distancing and broad social widespread social unrest stemming from the COVID-19 pandemic crisis has significantly impacted interpersonal relationships and led to substantial changes in lifestyle, employment, and social interactions^16^.

The pandemic has had far-reaching consequences, with people becoming socially distant, leading to many business closures and pushing millions below the poverty line^17^. As a result, many individuals are experiencing psychological distress, including symptoms of depression, anxiety, and post-traumatic stress^18^. Studies conducted during the pandemic have reported significant prevalence rates of COVID-19-related mental health conditions such as depression (15.97%), anxiety (15.15%), insomnia (23.87%), post-traumatic stress (21.94%), and psychological distress (13.29%)^19^. Healthcare professionals, in particular, had a significantly exhibited a significantly higher rate of insomnia at 36.52%^19^. Additionally, global research has indicated high levels of fear of COVID-19 due to the pandemic^20^. Furthermore, stringent disease containment measures adopted by governments, especially over prolonged periods, contribute to a phenomenon known as pandemic burnout. Pandemic burnout, characterized by emotional exhaustion and impacting various aspects of individuals' lives, has significant negative consequences on the trajectory of the pandemic. Individuals experiencing burnout may exhibit reduced adherence to protective health measures, leading to uncontrolled virus transmission, increased mortality rates, and prolonged adverse effects that perpetuate the ongoing pandemic^21^.

It is usual for people to experience stress and fear in response to infectious diseases, which can be maladaptive^22^. Fear is commonly observed in infectious disease outbreaks, and various factors can contribute to its development, such as fear of contracting or spreading an illness, losing loved ones, or severe restrictions on social interactions^23^. Prolonged and intense feelings of fear can have negative impacts on both mental and physical health. Therefore, gaining a better understanding of the reasons behind fear during pandemics can help minimize its impact in the future^24^.

The COVID-19 pandemic has highlighted the need to identify public health interventions that can prevent the spread of infectious diseases and mitigate social and economic impacts. Gaining insight into the psychological and behavioral patterns of individuals post-COVID-19 pandemic is of utmost importance. This understanding is critical for effectively implementing recommendations that promote adherence to guidelines and tailoring personalized intervention strategies. By doing so, we can effectively mitigate and reduce the negative health consequences that may arise in the future. Thus, the aim of this study is to understand the levels of fear experienced by individuals regarding future pandemics and/or epidemics among the general population of Cyprus and comprehensively examine the diverse factors, including but not limited to sociodemographic characteristics, factors related to health status, health literacy, trust, as well as specific factors related to the COVID-19 pandemic experience (e.g., previous infection, vaccination status, underlying health conditions), that influence this perceived fear. By investigating these factors, we aim to gain insights into the multifaceted determinants shaping the perception of fear and its associated factors among the population of Cyprus.

## Results

### Sociodemographic characteristics

A total of 1075 individuals participated in the study. The mean age of the participants was 43.6 years old (SD = 17.7 years old) with 13.2% (n = 142) aged 18–24 years old, 41.1% (n = 442) aged 25–44 years old, 30.3% (n = 326) aged 45–64 years old and 15.4% (n = 165) aged 65 years and over (Table 1). A total of 560 females (52.1%) participated in the study. Most of the participants were from Cyprus (n = 1019, 94.8%), residents of Nicosia (n = 429, 39.9%) and lived in urban areas (n = 920, 85.6%). In addition, the majority of the respondents were private employees (n = 391, 36.4%), were not healthcare professionals (n = 912, 86.9%), were married/in cohabitation (n = 648, 60.7%), had children (n = 616, 57.6%), had not underage children living in the household (n = 741, 70.0%), had completed an undergraduate education (n = 400, 37.3%) and had an annual income of more than €19,500 (n = 529, 49.4%) (Table 1).

**Table 1:** Sociodemographic characteristics overall and by fear of future epidemics and/or pandemics.

Characteristics	Overall (N = 1075)	Fear of future epidemics and/or pandemics		
		No (N = 498)	Yes (N = 577)	p-value
Mean age ± SD	43.6 ± 17.4	43.4 ± 17.1	43.7 ± 17.7	0.7796^g^
Age group, N^a^ (%)				
18–24	142 (13.2)	77 (54.2)	65 (45.8)	**0.001**^h^
25–44	442 (41.1)	176 (39.8)	266 (60.2)	
45–64	326 (30.3)	170 (52.2)	156 (47.8)	
65+	165 (15.4)	75 (45.5)	90 (54.5)	
Gender, N^a^ (%)				
Male	515 (47.9)	292 (56.7)	223 (43.3)	** < 0.001**^h^
Female	560 (52.1)	206 (36.8)	354 (63.2)	
Country, N^a^ (%)				
Cyprus	1019 (94.8)	472 (46.3)	547 (63.7)	0.953^h^
Greece	42 (3.9)	20 (47.6)	22 (52.4)	
Other	14 (1.3)	6 (42.9)	8 (57.1)	
Geographical area, N^a^ (%)				
Nicosia	429 (39.9)	191 (44.5)	238 (55.5)	**0.002**^h^
Limassol	297 (27.6)	141 (47.5)	156 (52.5)	
Larnaca	178 (16.6)	72 (40.5)	106 (59.5)	
Paphos	111 (10.3)	70 (63.1)	41 (36.9)	
Ammochostos	60 (5.6)	24 (40.0)	36 (60.0)	
Residency, N^a^ (%)				
Urban	920 (85.6)	433 (47.1)	487 (52.9)	0.236^h^
Rural	155 (14.4)	65 (41.9)	90 (58.1)	
Job status, N^a^ (%)				
Private employee	391 (36.4)	183 (46.8)	208 (53.2)	**0.013**^h^
State employee	274 (25.5)	112 (40.9)	162 (59.1)	
Freelance	114 (10.6)	66 (57.9)	48 (42.1)	
Unemployed	154 (14.3)	79 (51.3)	75 (48.7)	
Retired	142 (13.2)	58 (40.8)	84 (59.2)	
Healthcare status, N^b^ (%)				
No	912 (86.9)	428 (46.9)	484 (53.1)	0.836^h^
Yes	137 (13.1)	63 (46.0)	74 (54.0)	
Marital status, N^c^ (%)				
Unmarried	315 (29.5)	150 (47.6)	165 (52.4)	0.791^h^
Married/in cohabitation	648 (60.7)	294 (45.4)	354 (54.6)	
Separated/divorced/widowed	104 (9.8)	49 (47.1)	55 (52.9)	
Having children, N^d^ (%)				
No	454 (42.4)	213 (46.9)	241 (53.1)	0.792^h^
Yes	616 (57.6)	284 (46.1)	332 (53.9)	
Underage children living in the household, N^e^ (%)				
No	741 (70.0)	347 (46.8)	394 (53.2)	0.784^h^
Yes	318 (30.0)	146 (45.9)	172 (54.1)	
Educational level, N^f^ (%)				
Up to secondary education	321 (29.9)	137 (42.7)	184 (57.3)	0.238^h^
Undergraduate education	400 (37.3)	187 (46.8)	213 (53.2)	
Postgraduate education	352 (32.8)	173 (49.2)	179 (50.8)	
Annual income, N^d^ (%)				
Low (≤ €6500)	154 (14.4)	71 (46.1)	83 (53.9)	0.516^h^
Middle (€6500–19,500)	387 (36.2)	171 (44.2)	216 (55.8)	
High (> €19,500)	529 (49.4)	254 (48.0)	275 (52.0)	

SD; Standard deviation; ^a^N = 1075; ^b^N = 1049; ^c^N = 1067; ^d^N = 1070; ^e^N = 1059; ^f^N = 1073; ^g^Differences were tested using t-test; ^h^Differences were tested using chi^2^ test; Bold values indicate statistically significant association at 5% significance level.

The mean score of fear of future epidemics and/or pandemics was 17.4 (SD = 6.5, range: 8–40), indicating a moderate level of fear. As expected, participants who reported being afraid of future epidemics and/or pandemics had a higher score of fear (mean = 21.1, SD = 5.5) compared to those who did not report fear (mean = 13.1, SD = 4.6) (p < 0.001). Further information regarding the characteristics associated with fear of future epidemics and/or pandemics can be found in Supplementary Table 1. The largest statistically significant correlation estimate was found between perceived fear of future epidemics and/or pandemics and health literacy (p < 0.001). The greater statistically significant correlation estimate (r = 0.2883) refers to a small association between perceived fear of future epidemics and/or pandemics and health literacy (Supplementary Table 2).

### Perceived fear of future epidemics and/or pandemics and sociodemographic characteristics

Among the age groups 18–24 years old and 45–64 years old, a larger number of individuals reported as having no fear of future epidemics and/or pandemics while among the age groups 25–44 years old and 65 + years old a larger number of individuals reported as being afraid of future epidemics and/or pandemics (p = 0.001). In addition, more females reported as being afraid of future epidemics and/or pandemics compared to males (p < 0.001). We also found statistically significant associations between geographical area (p = 0.002) and job status (p = 0.013) among those who were afraid of future epidemics and/or pandemics and those who did not report fear (Table 1).

### Perceived fear of future epidemics and/or pandemics and COVID-19-related vaccination

We observed that the majority of respondents who were infected by COVID-19 (n = 818, 76.1%) did not belong to a vulnerable group (n = 876, 81.6%), did not have any chronic diseases (n = 760, 71.9%), and were vaccinated against COVID-19 (n = 918, 86.4%). Among those who received the COVID-19 vaccine, a significant portion had received 2 doses (n = 610, 66.0%) and had received the Pfizer/BioNTech vaccine (n = 625, 67.6%). Furthermore, nearly 30% of these participants did not have the intention to receive another dose if requested (n = 313), believed that the vaccine moderately helped to prevent COVID-19 disease (n = 77), and strongly believed that the vaccine reduces the symptoms of the disease (n = 200). On the other hand, among those who did not receive the COVID-19 vaccine, the majority (96.1%, n = 172) did not plan to receive it. Additionally, we found a higher percentage of individuals with fear of future epidemics and/or pandemics among participants who belonged to a vulnerable group (p = 0.015), had at least one chronic disease (p = 0.015), and were vaccinated against COVID-19 (p < 0.001). The most significant difference in fear levels was observed among those who expressed a very strong intention to receive another dose if requested (p < 0.001) and those who strongly believed that the vaccine reduces the symptoms of the disease (p = 0.012) (Supplementary Table 3).

### Perceived fear of future epidemics and/or pandemics and health status

More than 90% of the participants had no problems in walking about, in self-care and performing their usual activities. Also, most of the respondents had no pain or discomfort (n = 830, 77.7%) and they were moderately anxious or depressed (n = 526, 49.8%). We reported that most of the individuals who had no (n = 512, 52.6%) or some problems (n = 63, 64.3%) in walking about had also fear of future epidemics and/or pandemics (p = 0.048). Similarly, we found that most of the respondents who had no (n = 421, 50.7%) or moderate (n = 143, 62.2%) pain or discomfort (p = 0.002) as well as most of the participants who were moderately (n = 308, 58.6%) or extremely (n = 47, 71.2%) anxious or depressed (p < 0.001), reported as being afraid of future epidemics and/or pandemics. Also, individuals who had no fear of future epidemics and/or pandemics reported as having a higher visual analogue scale (mean = 76.1, SD = 54.0) compared to those who are afraid (mean = 65.1, SD = 31.8) (p < 0.001) (Supplementary Table 4).

### Perceived fear of future epidemics and/or pandemics and health literacy, trust in existing and future vaccine, in public health, science and medicine

The mean health literacy score of the participants is 7.9 (SD = 5.9, range: 0–16) with 51.7% (n = 556) had an inadequate health literacy status, 17.8% (n = 191) had a problematic health literacy status and 30.5% (n = 328) had an adequate health literacy (Supplementary Table 5). Moreover, we reported a higher score of health literacy (p < 0.001) among those who were afraid of future epidemics and/or pandemics (p < 0.001). Furthermore, the mean score in trust in existing and future vaccine, in public health, science, and medicine was 28.4 (SD = 7.6) with 27.9% (n = 300) having low trust, 23.4% (n = 252) having moderate trust, and 48.7% (n = 523) having high trust (Supplementary Table 6). A higher score of trust in existing and future vaccine, in public health, science and medicine, was reported among those who were afraid of future epidemics and/or pandemics (p = 0.0015). More details about health literacy and trust in existing and future vaccine overall as well as by fear of future epidemics and/or pandemics are presented in Supplementary Tables 5 and 6, respectively.

### Determinants of perceived fear of future epidemics and/or pandemics

As reported by the full model of the hierarchical logistic regression modelling (Model 4), sex, job status, educational level, health status, health literacy score, trust in public health, science and medicine score and COVID-19 vaccination status are related to the perceived fear of future epidemics and/or pandemics. Specifically, we discovered that females were more likely to be afraid of future epidemics and/or pandemics (OR = 2.37, 95% CI 1.78–3.16, p < 0.001) compared to males. On the other hand, we found that unemployed participants and those who had completed a postgraduate education were 70% (OR = 0.30, 95% CI 0.13–0.65, p = 0.003) and 44% (OR = 0.56, 95% CI 0.34–0.90, p = 0.016) less likely to be afraid of future epidemics and/or pandemics compared to private employees and individuals with secondary education, respectively. Furthermore, we reported that individuals with adequate health literacy (OR = 1.05, 95% CI 1.03–1.08, p < 0.001) and higher trust in public health, science, and medicine (OR = 1.04, 95% CI 1.02–1.06, p = 0.001) were more likely to be afraid of future epidemics and/or pandemics. Finally, we found that those who vaccinated against COVID-19 were 1.57 times more likely to be afraid of future epidemics and/or pandemics compared to those who did not vaccinate against COVID-19 (OR = 1.57, 95% CI 1.02–2.43, p = 0.041) (Table 2).

**Table 2:** Hierarchical logistic regression modelling on fear of future epidemics and/or pandemics (Yes vs. No).

Characteristics	Model 1		Model 2		Model 3		Model 4	
	OR (95% CI)	p-value	OR (95% CI)	p-value	OR (95% CI)	p-value	OR (95% CI)	p-value
Age	0.99 (0.97, 1.01)	0.335	0.99 (0.98, 1.01)	0.428	0.99 (0.97, 1.01)	0.291	0.99 (0.97, 1.00)	0.133
Sex								
Male	*Ref*		*Ref*		*Ref*		*Ref*	
Female	**2.28 (1.74, 3.00)**	** < 0.001**	**2.20 (1.67, 2.90)**	** < 0.001**	**2.29 (1.73, 3.05)**	** < 0.001**	**2.37 (1.78, 3.16)**	** < 0.001**
Country								
Cyprus	*Ref*		*Ref*		*Ref*		*Ref*	
Greece	1.13 (0.59, 2.16)	0.706	1.27 (0.66, 2.46)	0.472	1.20 (0.61, 2.35)	0.592	1.14 (0.58, 2.25)	0.697
Other	0.96 (0.31, 3.01)	0.947	0.96 (0.31, 3.03)	0.951	0.92 (0.29, 2.92)	0.887	0.93 (0.29, 2.96)	0.902
Geographical area								
Nicosia	*Ref*		*Ref*		*Ref*		*Ref*	
Limassol	0.94 (0.69, 1.30)	0.725	0.97 (0.70, 1.33)	0.831	0.98 (0.70, 1.37)	0.910	1.00 (0.71, 1.40)	0.989
Larnaca	1.20 (0.82, 1.77)	0.343	1.16 (0.79, 1.72)	0.440	1.19 (0.80, 1.76)	0.401	1.17 (0.78, 1.75)	0.448
Paphos	**0.48 (0.31, 0.77)**	**0.002**	**0.47 (0.30, 0.75)**	**0.002**	0.68 (0.42, 1.12)	0.131	0.70 (0.42, 1.17)	0.174
Ammochostos	1.43 (0.79, 2.62)	0.240	1.33 (0.72, 2.45)	0.359	1.24 (0.66, 2.30)	0.504	1.18 (0.63, 2.20)	0.610
Residency								
Urban	*Ref*		*Ref*		*Ref*		*Ref*	
Rural	1.07 (0.73, 1.57)	0.738	1.08 (0.73, 1.59)	0.704	1.05 (0.71, 1.56)	0.800	1.05 (0.71, 1.57)	0.797
Job status								
Private employee	*Ref*		*Ref*		*Ref*		*Ref*	
State employee	1.28 (0.90, 1.82)	0.173	1.27 (0.89, 1.81)	0.191	1.12 (0.78, 1.61)	0.542	1.11 (0.77, 1.61)	0.583
Freelance	0.78 (0.48, 1.26)	0.307	0.76 (0.47, 1.23)	0.268	0.77 (0.47, 1.25)	0.290	0.78 (0.48, 1.28)	0.328
Unemployed	**0.32 (0.15, 0.68)**	**0.003**	**0.32 (0.15, 0.70)**	**0.004**	**0.33 (0.15, 0.72)**	**0.005**	**0.30 (0.13, 0.65)**	**0.003**
Retired	1.32 (0.70, 2.48)	0.383	1.33 (0.70, 2.51)	0.383	1.10 (0.58, 2.12)	0.765	0.98 (0.50, 1.91)	0.945
Healthcare status								
No	*Ref*		*Ref*		*Ref*		*Ref*	
Yes	1.01 (0.68, 1.51)	0.957	1.00 (0.66, 1.50)	0.996	1.08 (0.71, 1.65)	0.724	1.05 (0.68, 1.61)	0.835
Marital status								
Unmarried	*Ref*		*Ref*		*Ref*		*Ref*	
Married/in cohabitation	1.03 (0.67, 1.57)	0.903	1.01 (0.66, 1.55)	0.962	1.04 (0.67, 1.60)	0.876	1.00 (0.64, 1.55)	0.993
Separated/divorced/widowed	0.79 (0.43, 1.45)	0.452	0.78 (0.42, 1.44)	0.429	0.81 (0.43, 1.51)	0.504	0.78 (0.42, 1.48)	0.454
Having children								
No	*Ref*		*Ref*		*Ref*		*Ref*	
Yes	0.91 (0.5, 1.63)	0.741	0.87 (0.48, 1.58)	0.645	0.92 (0.50, 1.68)	0.786	0.92 (0.50, 1.71)	0.798
Underage children living in the household								
No	*Ref*		*Ref*		*Ref*		*Ref*	
Yes	1.03 (0.67, 1.59)	0.897	1.02 (0.65, 1.58)	0.946	1.05 (0.67, 1.65)	0.826	1.11 (0.70, 1.76)	0.672
Educational level								
Up to secondary education	*Ref*		*Ref*		*Ref*		*Ref*	
Undergraduate education	**0.64 (0.43, 0.96)**	**0.030**	**0.62 (0.42, 0.94)**	**0.022**	**0.70 (0.46, 1.06)**	0.089	0.71 (0.46, 1.07)	0.104
Postgraduate education	**0.55 (0.35, 0.87)**	**0.010**	**0.54 (0.34, 0.85)**	**0.009**	**0.56 (0.35, 0.90)**	**0.016**	**0.56 (0.34, 0.90)**	**0.016**
Annual income								
Low (≤ €6500)	*Ref*		*Ref*		*Ref*		*Ref*	
Middle (€6500–19,500)	0.58 (0.28, 1.19)	0.138	0.59 (0.29, 1.23)	0.160	0.62 (0.30, 1.30)	0.204	0.60 (0.28, 1.26)	0.174
High (> €19,500)	0.57 (0.26, 1.24)	0.158	0.62 (0.28, 1.34)	0.223	0.65 (0.29, 1.44)	0.284	0.61 (0.27, 1.36)	0.225
Visual analogue scale	–	–	**0.99 (0.99, 1.00)**	**0.002**	**0.99 (0.99, 1.00)**	**0.003**	0.99 (0.99, 1.00)	**0.009**
Health literacy score	–	–	–	–	**1.06 (1.03, 1.09)**	** < 0.001**	**1.05 (1.03, 1.08)**	** < 0.001**
Trust score	–	–	–	–	**1.05 (1.02, 1.07)**	** < 0.001**	**1.04 (1.02, 1.06)**	**0.001**
Vulnerable group								
No/I am not sure	*Ref*		*Ref*		*Ref*		*Ref*	
Yes	–	–	–	–	–	–	1.11 (0.69, 1.78)	0.669
Having at least one chronic disease								
No	*Ref*		*Ref*		*Ref*		*Ref*	
Yes	–	–	–	–	–	–	1.41 (0.94, 2.13)	0.094
Covid-19 vaccine								
No	*Ref*		*Ref*		*Ref*		*Ref*	
Yes	–	–	–	–	–	–	**1.57 (1.02, 2.43)**	**0.041**

OR, odds ratio; CI, confidence interval; Model 1: Sociodemographic characteristics on fear of future epidemics and/or pandemics (Yes vs. No); Model 2: Sociodemographic characteristics and visual analogue scale on fear of future epidemics and/or pandemics (Yes vs. No); Model 3: Sociodemographic characteristics, visual analogue scale, health literacy score and trust score on fear of future epidemics and/or pandemics (Yes vs. No); Model 4: Sociodemographic characteristics, visual analogue scale, health literacy score, trust score, being in a vulnerable group, having on fear of future epidemics and/or pandemics (Yes vs. No); Bold values indicate statistically significant association at 5% significance level.

Linear regression model indicated that increased age (β = − 0.10, 95% CI − 0.14, − 0.05) was negatively associated with a higher score of fear of future epidemics and/or pandemics. In addition, being female (β = 2.68, 95% CI 1.91, 3.46), living in Larnaca (β = 1.25, 95% CI 0.70, 2.33), having a higher health literacy score (β = 0.25, 95% CI 0.18, 0.32), being in a vulnerable group (β = 2.02, 95% CI 0.75, 3.28), having at least one chronic disease (β = 1.76, 95% CI 0.68, 2.84) and receiving a COVID-19 vaccine (β = 2.12, 95% CI 0.96, 3.28) were positively associated with a higher score of fear of future epidemics and/or pandemics (Table 3).

**Table 3:** Linear regression modelling on fear of future epidemics and/or pandemics (range: 8–40, maximum score indicates higher fear).

Characteristics	β-coefficient (95% CI)	p-value
Age	**− 0.10 (− 0.14, − 0.05)**	** < 0.001**
Sex		
Male	*Ref*	
Female	**2.68 (1.91, 3.46)**	** < 0.001**
Country		
Cyprus	*Ref*	
Greece	− 1.22 (− 3.07, 0.62)	0.192
Other	− 0.35 (− 3.57, 2.86)	0.829
Geographical area		
Nicosia	*Ref*	
Limassol	0.01 (− 0.91, 0.92)	0.986
Larnaca	**1.25 (0.70, 2.33)**	**0.024**
Paphos	− 0.69 (− 2.04, 0.66)	0.316
Ammochostos	1.36 (− 0.31, 3.02)	0.109
Residency		
Urban	*Ref*	
Rural	− 0.05 (− 1.13, 1.02)	0.922
Job status		
Private employee	*Ref*	
State employee	0.08 (− 0.92, 1.08)	0.877
Freelance	− 0.51 (− 1.85, 0.83)	0.457
Unemployed	− 0.56 (− 2.60, 1.48)	0.588
Retired	1.39 (− 0.40, 3.17)	0.129
Healthcare status		
No	*Ref*	
Yes	− 0.13 (− 1.28, 1.03)	0.829
Marital status		
Unmarried	*Ref*	
Married/in cohabitation	− 0.03 (− 1.23, 1.17)	0.961
Separated/divorced/widowed	− 1.51 (− 3.20, 0.19)	0.081
Having children		
No	*Ref*	
Yes	0.37 (− 1.28, 2.02)	0.664
Underage children living in the household		
No	*Ref*	
Yes	0.12 (− 1.13, 1.36)	0.854
Educational level		
Up to secondary education	*Ref*	
Undergraduate education	0.11 (− 1.03, 1.24)	0.852
Postgraduate education	− 0.77 (− 2.06, 0.52)	0.240
Annual income		
Low (≤ €6500)	*Ref*	
Middle (€6500–19,500)	− 0.50 (− 2.37, 1.38)	0.604
High (> €19,500)	− 0.30 (− 2.35, 1.74)	0.770
Visual analogue scale	− 0.01 (− 0.02, 0.00)	0.065
Health literacy score	**0.25 (0.18, 0.32)**	** < 0.001**
Trust score	0.01 (− 0.05, 0.07)	0.707
Vulnerable group		
No/I am not sure	*Ref*	
Yes	**2.02 (0.75, 3.28)**	**0.002**
Having at least one chronic disease		
No	*Ref*	
Yes	**1.76 (0.68, 2.84)**	**0.001**
Covid-19 vaccine		
No	*Ref*	
Yes	**2.12 (0.96, 3.28)**	** < 0.001**

CI, confidence interval; Bold values indicate statistically significant association at 5% significance level.

## Discussion

This study represents the first attempt to investigate the influence of the COVID-19 pandemic on the perceived fear of future pandemics and/or epidemics. Its primary objective is to understand the level of fear experienced by individuals and identify associated factors. Based on our research, 53.7% of the participants expressed fear regarding future pandemics and/or epidemics. It is noteworthy that women and individuals who have received COVID-19 vaccinations are more likely to report fear. Additionally, being in a vulnerable group or having at least one chronic disease, along with higher levels of health literacy and trust in existing and future vaccines, public health, science, and medicine, are associated with an increased likelihood of fearing future pandemics. Conversely, older age, unemployment, and having a master's degree are associated with a lower probability of perceived fear.

According to our findings, gender emerged as one of the strongest factors associated with perceived fear of future pandemics and/or epidemics among the general population of Cyprus. Our results demonstrated a higher likelihood of females experiencing fear of future epidemics and/or pandemics compared to males. This finding aligns with recent studies conducted in the United States of America, Cuba, and China, which consistently reported greater levels of fear and stress of COVID-19 during pandemic among women^25–28^. Notably, a previous meta-analysis indicated that the COVID-19 pandemic had a significantly greater negative impact on women, particularly in terms of higher levels of fear and anxiety. The gender disparity in worry and fear was even more pronounced in Europe^29^. The observed increase in fear among women in our study is consistent with the general tendency for women to experience negative emotions, including fear, more frequently than men^30^. This heightened fear in women could be attributed to their stronger emotional experiences and relatively higher vulnerability compared to men^31^. Additionally, women have been found to have higher rates of psychological disorders associated with anxiety and fear compared to men^32^.

People with higher levels of health literacy were also found to be more likely to experience fear of future pandemics and/or epidemics. However, our findings contradict the existing literature on fear of COVID-19 during the COVID-19 pandemic in relation to health literacy. Multiple studies have consistently shown that increased health literacy is associated with reduced fear of COVID-19^33–37^. The discrepancy between our results and the existing literature may be attributed to the fact that the previous studies focus on the well-documented COVID-19 pandemic, where there is a wealth of information available. Consequently, highly literate individuals perceive and understand the risks and relevant information surrounding the pandemic. In contrast, fear of a future pandemic arises from the unknown, and it is this uncertainty that may contribute to the fear experienced. In light of this, individuals with higher literacy are more likely to fear future pandemics due to the uncertainty caused by the COVID-19 pandemic at its onset. These individuals possess the ability to seek, comprehend, and utilize health-related information when it is available, which is not the case for future situations.

People who have received the COVID-19 vaccination appear to have a higher likelihood of experiencing fear of future pandemics. This finding is consistent with previous studies that have examined the relationship between vaccination, fear of the COVID-19 pandemic, and mental health problems. Various studies conducted worldwide have consistently shown a positive association between heightened fear of the pandemic and increased acceptance of vaccination^38–41^. Similarly, research has shown that individuals experiencing mental health issues tend to exhibit higher acceptance and willingness to vaccinate^42^. The most plausible explanation for this association is that individuals tend to avoid what they fear. Therefore, the increased fear of pandemics and vaccination can be attributed to people's willingness to be vaccinated to mitigate the perceived threat of COVID-19 and, more broadly, future pandemics. The specific fear arises from the realization that a pandemic and/or epidemic poses a threat to their own lives and the well-being of their communities. Consequently, individuals are expected to be more receptive to a solution to this problem, which, in the case of COVID-19 and this research, is vaccination.

Our findings also revealed a significant observation: there is a positive relationship between the level of trust in existing and future vaccines, public health, science, and medicine, and the fear of future pandemics. Although no direct comparisons are available for similar results, this finding can potentially be explained by the fact that individuals who have trust in science, medicine, vaccines, as well as the decisions made by government and public health authorities, may experience heightened anxiety and fear regarding the pandemic. These individuals are likely to proactively follow guidelines and instructions provided to them, as they prioritize their health, which in turn may contribute to an increased fear of future pandemics. Previous studies suggest that higher levels of trust in science were negatively correlated with COVID-19 vaccine hesitancy^43,44^. Further research is needed to elucidate the underlying factors and mechanisms that contribute to our observed correlations.

Our findings also indicated that individuals with at least one chronic condition were nearly twice as likely to report fear of future pandemics or epidemics compared to those without any chronic conditions. These findings align with expectations, as individuals with chronic conditions are more vulnerable in terms of their health, especially in situations of potential pandemics where the risks of complications, morbidity, and mortality are elevated^45^. Similar results have been highlighted in a study conducted during the COVID-19 pandemic where individuals with comorbidities reported significantly higher levels of fear of COVID-19^46^. Likewise, participants belonging to vulnerable groups were more than twice as likely to report fear of future pandemics compared to those not in vulnerable groups. Several studies conducted during the COVID-19 pandemic have consistently demonstrated that individuals with underlying diseases and those belonging to increased risk groups exhibit higher levels of fear^46–49^. These findings are in line with expectations, considering that individuals with diseases and belonging to vulnerable groups face an elevated risk to their health during epidemic or pandemic situations.

Our findings reveal a negative association between participant age and perceived fear. Prior studies have presented diverse results concerning the relationship between age and fear of COVID-19, with some indicating an increase in fear with age^46,50,51^, while others suggest higher fear among younger adults^52,53^. However, a recent systematic review and meta-analysis demonstrated no significant association between the average fear of COVID-19 and participants' age^20^. The disparity in findings could be attributed to the specific focus of our study, which examines fear related to potential future pandemics or epidemics rather than the current COVID-19 situation. Younger individuals may harbor concerns about their future, encompassing potential socio-economic consequences and health impacts, recognizing their likelihood of experiencing such events in the near or distant future. Conversely, older individuals may be less apprehensive about future pandemics, assuming that they may not be personally affected by them.

Our findings indicated that unemployed participants had a lower likelihood of experiencing fear of future epidemics and/or pandemics compared to private employees. One possible explanation for these results is that fear often stems from job uncertainty, especially during unfavorable economic conditions worldwide, as observed during the pandemic. Unemployed individuals, particularly those who are jobless by choice, may be less prone to anxiety related to potential job loss. However, it is important to note that these findings contradict existing but limited literature. For instance, a recent study has shown that unemployment tends to increase the likelihood of fear related to COVID-19^54^. Our findings further revealed that individuals with postgraduate education had a lower likelihood of experiencing fear of future epidemics and/or pandemics compared to individuals with secondary education. This aligns with previous research studies that have shown a similar trend. For instance, a previous study aiming to identify factors influencing fear of infection by COVID-19 during the current pandemic found that individuals with higher levels of education exhibited less fear of being infected compared to those with lower levels of education^55^. Consistent results were also observed in a cross-sectional study conducted in Greece, where participants with higher education reported lower levels of fear on average^46^. Conversely, research conducted in Bangladesh demonstrated opposite results, with participants with higher education reporting greater fear of COVID-19 compared to those with lower levels of education^56^. The findings of our study regarding the inverse relationship between higher education and reduced likelihood of fear may be attributed to the fact that individuals with education and knowledge possess a better understanding of the overall pandemic situation and are less influenced by false information or misinformation circulating in mass media or on the internet, which often contribute to instilling fear in the population.

The findings of this research have significant implications for public health. The fact that more than half of the participants (53.7%) reported fear of future pandemics and/or epidemics highlights the urgent need for concrete measures and programs to reduce this rate and promote optimal mental health in the population. Prior research underscores the significance of enhancing community and subnational cluster capacity, tackling health and socioeconomic disparities, and establishing collaborative multi-sectoral mechanisms to maximize COVID-19 control endeavors^57^. Given the wide reach of these results across the general population of Cyprus, the substantial percentage of fear undoubtedly calls for the attention of relevant authorities to improve the situation. This can be achieved through increased public information and enhanced health literacy, particularly considering that more than half of the population demonstrated insufficient literacy. However, it is important to note that increased literacy in this study was associated with increased fear. Nevertheless, in order to counteract fear and anxiety, and explicitly tackle the misinformation and disinformation within the infodemic, it is crucial to disseminate correct information related to the health impacts of infectious diseases causing high global public concerns. By doing so, we can empower individuals with accurate knowledge, enabling them to make informed decisions and alleviate unfounded fears. Instead, the focus should be on providing simple and accessible information to improve perception and knowledge. Special emphasis should be placed on the dissemination of information through mass media, as individuals who place high trust in them tend to exhibit significantly increased fear of future pandemics. News and related information should be presented in a balanced and sensitive manner to avoid causing unnecessary panic among the public. Furthermore, vulnerable groups with chronic diseases and individuals experiencing moderate to severe pain in their daily lives were found to be at a significantly higher risk of experiencing fear. This underscores the need for additional support, particularly psychological support, for these vulnerable groups within society. Health professionals such as doctors, nurses, and pharmacists, who frequently interact with these individuals, can play a crucial role in providing this support. Additionally, offering free psychological support from psychologists can be an effective approach. Lastly, considering the finding that women experience greater fear compared to men, there is a clear need for increased psychological support tailored to the needs of women during such situations.

However, it is important to acknowledge the limitations of the present study, which should be considered when interpreting the results. Firstly, the use of self-report questionnaires to collect data introduces the potential for information bias, which is a common issue in cross-sectional studies. However, the nature of the concepts of interest in this study primarily relies on self-report measures. Moreover, the participant recruitment method and data collection may limit the representativeness of the study. Furthermore, the fact that the questionnaire was exclusively available in the Greek language partially restricts the sample to individuals who are proficient in Greek, excluding permanent residents of Cyprus who do not possess this proficiency. Finally, it is important to note that these findings are specific to the residents of Cyprus and may not be readily generalized to other countries. Further research is necessary to investigate the fear of potential future pandemics and/or epidemics in diverse populations, including countries with different cultural backgrounds. Additionally, exploring the role of other characteristics, such as psychological, personal, temperamental, and contextual factors, could provide valuable insights. Conducting studies that incorporate these suggestions would contribute to a more comprehensive understanding of fear in the context of future pandemics and/or epidemics..

## Conclusions

This study represents the first attempt to examine the levels of fear experienced by individuals regarding future pandemics and/or epidemics among the general population of Cyprus and explore the diverse factors influencing this perceived fear. Our findings reveal that more than half of the participants reported experiencing fear of future pandemics and/or epidemics, with women and individuals vaccinated against COVID-19 displaying significantly higher levels of fear. Moreover, being in a vulnerable group or having at least one chronic disease, along with higher health literacy and greater trust in vaccines, public health, science, and medicine were associated with an increased likelihood of reporting fear. Conversely, older age, unemployment, and having a master's degree are associated with a lower probability of perceived fear. It is imperative for the government of Cyprus and relevant authorities to prioritize mental health and disseminate information in a manner that avoids spreading fear and panic, particularly among vulnerable population groups. Future studies should replicate these findings, contributing to a comprehensive understanding of predictive factors influencing fear for effective global response.

## Methods

### Study design

This was a cross-sectional survey. During the data collection period, there were no limitations on mobility, allowing citizens to access a wide range of services and amenities, without the necessity of presenting any health certificates. In some public places, wearing face masks was required. This study was reported following the Strengthening the Reporting of Observational Studies in Epidemiology^58^.

### Study population

The referent population included Greek-Cypriot men and women aged 18 years old and above living in the five government-controlled municipalities of the Republic of Cyprus (Nicosia, Limassol, Larnaca, Paphos and Ammochostos) who could read and understand Greek language. This study was carried out following the Helsinki Declaration guidelines and was approved by the Cyprus National Bioethics Committee (ΕΕΒΚ ΕΠ 2022.01.206). The study has not received any funding. Informed consent was obtained from all participants prior to the start of the data collection.

### Sampling and sample size

Sample recruitment took place between October 1st, 2022, to February 19th, 2023, through a combination of face-to-face administration of the questionnaires in public crowded places (e.g., restaurants, malls, cafes, and main squares) and the use of instant messaging apps, such as WhatsApp, Viber, Facebook Messenger. A proportional quota sampling was used for the recruitment, a nonprobability sampling strategy to improve the representation of underrepresented groups in the survey by recruiting a fixed number of participants from each age group, sex, and place of residence^59^. This method was used to mitigate potential selection bias, to represent the study population according to the five government-controlled districts of the Republic of Cyprus, as reported by the Statistical Service of Cyprus Nicosia (39%), Limassol (28%), Larnaca (17%), Paphos (10%), and Ammochostos (6%)]^60^. The required sample size to estimate the percentage of participants answering ‘yes’ to the question of whether they are afraid, worried, or insecure about the possibly of future epidemics and/or pandemics of communicable diseases threatening human health was 1070. Specifically, using a 95% confidence interval (CI) and assuming a precision of 5% with a true prevalence of fear about the possibility of future epidemics and/or pandemics of communicable diseases threatening human health of 50% in the population, the sample size required for the study was calculated to be 1070. The final study sample did not significantly differ from the general population in terms of age, sex, and geographical area (all p-values > 0.05).

### Study instrument and measures

Data were collected by a self-administered anonymous questionnaire, which was developed by researchers based on previous experience and an extensive literature search. The questionnaire contained 68 closed-ended and multiple-choice questions written in Greek. Some parts of the questionnaire consisted of validated questionnaires that had been previously validated in Greek, for which relevant permission was obtained. The final questionnaire was validated through a pilot study prior to the actual study, in which 25 participants were recruited to assess the clarity and applicability of all survey items and to identify any wording problems that may have occurred during the actual data collection. These participants were not included in the study sample.

#### Sociodemographic characteristics

The questionnaire contained 13 questions about sociodemographic characteristics. Sex was reported as men, women or other, while age was reported in years. Origin country was either Cyprus, Greece or other, while the city of residence included the five districts of Cyprus, and area of living was recorded as urban or rural. Occupation was recorded as a private or state employee, freelancer, student, unemployed, housewife, or retired. Healthcare professionals were identified through a binary question (Yes vs. No), and if the answer was “Yes”, participants were asked to select their profession from a list that included nurse, doctor, dentist, pharmacist, psychologist, physiotherapist, or the option to report other. Marital status was recorded as married, living with a partner, single, separated, divorced, or widowed. The presence of children was evaluated through a binary question (Yes/No), and if the answer was “Yes”, participants were asked whether their children were underage. The educational level was divided into three categories: (i) those who completed up to high school, (ii) those who had a college or university degree, and (iii) those who had a master’s or PhD degree. Income was categorized into three groups as follows: (i) low (≤ €6500), (ii) moderate (€6500–19,500), and (iii) high (> €19,500) on an annual basis.

#### Health status

The health-related quality of life was measured using the EQ-5D-3L questionnaire^61^. The questionnaire consists of six questions, divided into two parts. The first part, called the descriptive system, consists of five three-level health dimensions: mobility, self-care, usual activities, pain/discomfort, and anxiety/depression. Respondents are asked to rate their level of difficulty in each dimension using three-level responses: no problems, some problems, or too many problems. The second part of the questionnaire asks respondents to rate their overall health on a vertical scale (vertical visual analogue scale) that ranges from “the best state of health you can imagine” to “the worst state of health you can imagine”.

#### Health literacy

The validated HLS-EU-Q16 questionnaire was used to measure the level of health literacy of the participants^62^. The questionnaire includes 16 questions formulated on a Likert scale (ranging from “Very Difficult” = 1 to “Very Easy” = 4) with self-report questions regarding difficulties in tasks related to decision-making in healthcare, disease prevention, and health promotion. For example: “On a scale from very easy to very difficult, how easy would you say it is to find information on treatments for illnesses that concern you?”. The HLS-EU-Q16 manual refers to dichotomizing the answers from HLS-EU-Q16 as ‘very difficult’/‘fairly difficult’ = 0 to ‘fairly easy’/‘very easy’ = 1 (range: 0–16). Higher scores indicate adequate health literacy. Based on that score, we defined three levels of health literacy as: inadequate (0–8), problematic (9–12) and adequate (13–16)^62^.

#### Trust in vaccines, public health authorities, science, and medicine

This part consists of 10 questions that concern the participants’ trust in existing vaccines (non-COVID-19 and COVID-19 vaccines) and future vaccines, trust in government and public health authorities regarding health issues, science, medical community, other healthcare professionals, as well as trust in media and influential people outside the vaccination system such as friends and family regarding health issues with all items scored on a 5-point Likert scale, ranging from 1 (no trust) to 5 (a lot of trust). We calculated the trust score in existing and future vaccine, in public health, science and medicine by adding the points of each of the 10 trust items (maximum score 50). Higher scores indicate higher trust. The Cronbach’s α-value for internal reliability was 0.9034. The tertiles of the score in trust were estimated, dividing the sample into three subgroups as follows: low trust (≤ 23), moderate trust (24–28) and high trust (≥ 29).

#### COVID-19 and vaccination against it

This part of the questionnaire contains 14 questions that pertain to COVID-19, including questions about the disease caused by the virus, whether the participant has any chronic diseases, and where they obtain information related to COVID-19. This section is further divided into questions for vaccinated participants and questions for non-vaccinated participants. For vaccinated participants, the following questions are included: (i) number of vaccine doses received, (ii) type of vaccine received, (iii) willingness to receive an additional dose, (iv) perceived effectiveness of the vaccine in preventing disease (for participants who have not been infected), (v) perceived effectiveness of the vaccine in reducing symptoms (for participants who have been infected), and (vi) reasons for getting vaccinated. For non-vaccinated participants, the following questions are included: (i) reasons for not getting vaccinated, and (ii) intention to get vaccinated in the future.

#### Perceived fear of future epidemics and/or pandemics

The first question of this part was evaluated using a binary question (Yes/No) regarding the fear, worry or insecurity about the possibility of future epidemics and/or pandemics of communicable diseases threatening human health. In addition, this part consists of 9 questions that assess fear, worry or insecurity of future pandemics and/or epidemics, as well as the intensity of those feelings, provided using the Likert scale (absolutely disagree = 1 to absolutely agree = 5) based on a validated questionnaire^63^. We calculated the score in fear of future epidemics and/or pandemics by adding the points of each of the 9 items (maximum score 45). Higher scores indicate lower fear of future epidemics and/or pandemics. The Cronbach’s α-value for internal reliability was 0.9147.

### Statistical analysis

The Shapiro–Wilk test was utilized to assess the normal distribution of quantitative variables. Sociodemographic characteristics of the participants were presented as mean ± standard deviation (SD) for continuous variables and as absolute (n) and relative frequencies (%) for categorical variables. The association between the categorical characteristics included in the study and the binary variable ‘fear of future epidemics and/or pandemics’ (Yes vs. No) was examined using the chi-square test of independence. Furthermore, the differences in the quantitative variables included in the study between the categories of the binary variable ‘fear of future epidemics and/or pandemics’ (Yes vs. No) were evaluated using the independent sample t-test.

Correlations between included measures (health status, health literacy, trust in vaccines, public health authorities, science, medicine, and perceived fear of future epidemics and/or pandemics) were examined using Pearson’s coefficient. Hierarchical logistic regression analysis was used to examine the association between sociodemographic characteristics, health status, health literacy, trust in vaccines, public health authorities, science, and medicine, and COVID-19 related characteristics on the perceived fear of future epidemics and/or pandemics (Yes vs. No) as a dependent variable. Firstly, we added the sociodemographic characteristics (Model 1), and after the health status characteristic (visual analogue scale) was added (Model 2). Then, we added the health literacy score and trust in vaccines, public health authorities score (Model 3), and finally, COVID-19 related characteristics were added (Model 4). In addition, a linear regression model was applied to examine the association between sociodemographic characteristics, health status, health literacy, trust in vaccines, public health authorities, science, and medicine, and COVID-19 related characteristics on the perceived fear of future epidemics and/or pandemics (continuous variable) as a dependent variable. All statistical tests performed were two‐sided, with the statistical significance level set at α = 0.05. The statistical analysis was conducted using STATA 14.0 (Stata Corp.).

## Supplementary Information

Supplementary Tables.

## Data Availability

The datasets used and/or analyzed during the current study are available from the corresponding author on reasonable request.
