# Supplementary material for: Factors associated with perceived fear of future pandemics and/or epidemics: a cross-sectional study in Cyprus
Source: Sci Rep. 2023 Jul 27;13:12194. doi: 10.1038/s41598-023-39381-2 (PMC10374585; doi:10.1038/s41598-023-39381-2)
Supplement: Supplementary file 1 — Supplementary Tables. [file 41598_2023_39381_MOESM1_ESM.docx]

**Supplementary Table 1.** Fear of pandemic-related characteristics overall and by levels of fear of future epidemics and/or pandemics.

|  | **Overall** (N=1075) | **Fear of future epidemics or pandemics** | | |
| --- | --- | --- | --- | --- |
|  |  | **No** (N=498) | **Yes** (N=577) | **p-value** |
| **I am fearful of the possibility of future epidemics and/or pandemics of communicable diseases threatening human health**, N^a^ (%) | | | | |
| Absolutely disagree | 178 (16.6) | 170 (95.5) | 8 (4.5) | **<0.001^c^** |
| Disagree | 212 (19.7) | 173 (81.6) | 39 (18.4) |  |
| Neither agree nor disagree | 346 (32.2) | 138 (39.9) | 208 (60.1) |  |
| Agree | 291 (27.0) | 15 (5.2) | 276 (94.8) |  |
| Absolutely agree | 48 (4.5) | 2 (4.2) | 46 (95.8) |  |
| **When I think about the possibility of future epidemics and/or pandemics of communicable diseases threatening human health, I feel insecure**, N^a^ (%) | | | | |
| Absolutely disagree | 194 (18.0) | 176 (90.7) | 18 (9.3) | **<0.001^c^** |
| Disagree | 192 (17.9) | 162 (84.4) | 30 (15.6) |  |
| Neither agree nor disagree | 308 (28.6) | 123 (39.9) | 185 (60.1) |  |
| Agree | 333 (31.0) | 37 (11.1) | 296 (88.9) |  |
| Absolutely agree | 48 (4.5) | 0 (0.0) | 48 (100.0) |  |
| **I am very concerned about the possibility of future epidemics and/or pandemics of communicable diseases threatening human health**, N^a^ (%) | | | | |
| Absolutely disagree | 197 (18.3) | 176 (89.3) | 21 (10.7) | **<0.001^c^** |
| Disagree | 201 (18.7) | 166 (82.6) | 35 (17.4) |  |
| Neither agree nor disagree | 319 (29.7) | 132 (41.4) | 187 (58.6) |  |
| Agree | 313 (29.1) | 24 (7.7) | 289 (92.3) |  |
| Absolutely agree | 45 (4.2) | 0 (0.0) | 45 (100.0) |  |
| **My hands sweat when I think about the possibility of future epidemics and/or pandemics of communicable diseases threatening human health**, N^a^ (%) | | | | |
| Absolutely disagree | 521 (48.5) | 329 (63.2) | 192 (36.8) | **<0.001^c^** |
| Disagree | 335 (31.2) | 124 (37.0) | 211 (63.0) |  |
| Neither agree nor disagree | 172 (16.0) | 44 (25.6) | 128 (74.4) |  |
| Agree | 35 (3.2) | 1 (2.9) | 34 (97.1) |  |
| Absolutely agree | 12 (1.1) | 0 (0.0) | 12 (100.0) |  |
| **I have difficulty concentrating because of my concern about the possibility of future epidemics and/or pandemics of communicable diseases threatening human health**, N^a^ (%) | | | | |
| Absolutely disagree | 609 (56.6) | 364 (59.8) | 245 (40.2) | **<0.001^c^** |
| Disagree | 306 (28.5) | 111 (36.3) | 195 (63.7) |  |
| Neither agree nor disagree | 120 (11.2) | 23 (19.2) | 97 (80.8) |  |
| Agree | 32 (3.0) | 0 (0.0) | 32 (100.0) |  |
| Absolutely agree | 8 (0.7) | 0 (0.0) | 8 (100.0) |  |
| **I have difficulty sleeping because of my concern about the possibility of future epidemics and/or pandemics of communicable diseases threatening human health**, N^a^ (%) | | | | |
| Absolutely disagree | 646 (60.1) | 374 (57.9) | 272 (42.1) | **<0.001^c^** |
| Disagree | 288 (26.8) | 103 (35.8) | 185 (64.2) |  |
| Neither agree nor disagree | 115 (10.7) | 21 (18.3) | 94 (81.7) |  |
| Agree | 23 (2.1) | 0 (0.0) | 23 (100.0) |  |
| Absolutely agree | 3 (0.3) | 0 (0.0) | 3 (100.0) |  |
| **When I see news about the possibility of future outbreaks and/or pandemics of communicable diseases on social media, I get anxious or nervous**, N^b^ (%) | | | | |
| Absolutely disagree | 387 (36.1) | 285 (73.6) | 102 (26.4) | **<0.001^c^** |
| Disagree | 275 (25.6) | 138 (50.2) | 137 (49.8) |  |
| Neither agree nor disagree | 226 (21.1) | 53 (23.5) | 173 (76.5) |  |
| Agree | 163 (15.2) | 21 (12.9) | 142 (87.1) |  |
| Absolutely agree | 22 (2.0) | 0 (0.0) | 22 (100.0) |  |
| **I feel my heartbeat faster or my heart rate increase when I think about the possibility of future outbreaks and/or pandemics of communicable diseases on social media, I get nervous or anxious**, N^b^ (%) | | | | |
| Absolutely disagree | 561 (52.3) | 341 (60.8) | 220 (39.2) | **<0.001^c^** |
| Disagree | 295 (27.5) | 122 (41.4) | 173 (58.6) |  |
| Neither agree nor disagree | 160 (14.9) | 30 (18.8) | 130 (81.2) |  |
| Agree | 50 (4.7) | 4 (8.0) | 46 (92.0) |  |
| Absolutely agree | 7 (0.6) | 0 (0.0) | 7 (100.0) |  |
| **Mean score of fear of future epidemics or pandemics*** (8-40) ± SD | 17.4 ± 6.5 | 13.1 ± 4.6 | 21.1 ± 5.5 | **<0.001^d^** |
| Abbreviations: SD; Standard Deviation; ^a^N=1075; ^b^N=1075; ^c^Differences were tested using chi2 test; ^d^Differences were tested using t-test; Bold values indicate statistically significant association at 5% significance level. Maximum score indicates higher fear. | | | | |

**Supplementary Table 2.** Correlation matrix of all included measures.

|  | **Health status** | **Health literacy** | **Trust in vaccines, public health authorities, science, medicine** | **Perceived fear of future epidemics or pandemics** |
| --- | --- | --- | --- | --- |
| **Health status** | 1 |  |  |  |
| **Health literacy** | -0.1104* | 1 |  |  |
| **Trust in vaccines, public health authorities, science, medicine** | 0.1319* | -0.1414* | 1 |  |
| **Perceived fear of future epidemics or pandemics** | -0.1463* | 0.2883* | -0.0131 | 1 |
| *Indicates statistically significant association at 1% significance level. | | | | |

**Supplementary Table 3.** Information about participants’ COVID-19 vaccination overall and by fear of future epidemics and/or pandemics.

|  | **Overall** (N=1075) | **Fear of future epidemics or pandemics** | | |
| --- | --- | --- | --- | --- |
|  |  | **No** (N=498) | **Yes** (N=577) | **p-value**^k^ |
| **Covid-19 infection**, N^a^ (%) | | | | |
| No/Do not know | 257 (23.9) | 123 (47.9) | 134 (52.1) | 0.572 |
| Yes | 818 (76.1) | 375 (45.8) | 443 (54.2) |  |
| **Vulnerable group**, N^b^ (%) | | | | |
| No/I am not sure | 876 (81.6) | 422 (48.2) | 454 (51.8) | **0.015** |
| Yes | 197 (18.4) | 76 (38.6) | 121 (61.4) |  |
| **Having at least one chronic disease**, N^c^ (%) | | | | |
| No | 760 (71.9) | 373 (49.1) | 387 (50.9) | **0.006** |
| Yes | 297 (28.1) | 118 (39.7) | 179 (60.3) |  |
| **COVID-19 vaccine**, N^d^ (%) | | | | |
| No | 144 (13.6) | 89 (61.8) | 55 (38.2) | **<0.001** |
| Yes | 918 (86.4) | 403 (43.9) | 515 (56.1) |  |
| **Number of doses**, N^e^ (%) | | | | |
| 0 | 9 (1.0) | 6 (66.7) | 3 (33.3) | **<0.001** |
| 1 | 207 (22.4) | 109 (52.7) | 98 (47.3) |  |
| 2 | 610 (66.0) | 266 (43.6) | 344 (56.4) |  |
| 3 | 91 (9.8) | 20 (22.0) | 71 (78.0) |  |
| 4 | 8 (0.8) | 4 (50.0) | 4 (50.0) |  |
| **Type of COVID-19 vaccine**, N^f^ (%) | | | | |
| Pfizer/BioNTech | 625 (67.6) | 275 (44.0) | 350 (56.0) | 0.153 |
| AstraZeneca | 65 (7.0) | 25 (38.5) | 40 (61.5) |  |
| Moderna | 105 (11.4) | 43 (41.0) | 62 (59.0) |  |
| Johnson & Johnson | 20 (2.2) | 14 (70.0) | 6 (30.0) |  |
| Combination | 108 (11.8) | 48 (44.0) | 61 (56.0) |  |
| **Intention to receive another dose if requested**, N^g^ (%) | | | | |
| Not at all | 313 (33.4) | 163 (52.1) | 150 (47.9) | **<0.001** |
| Little | 203 (21.6) | 97 (47.8) | 106 (52.2) |  |
| Moderate | 192 (20.5) | 66 (34.4) | 126 (65.6) |  |
| A lot | 137 (14.6) | 55 (40.2) | 82 (59.8) |  |
| Very much | 93 (9.9) | 31 (33.3) | 62 (66.7) |  |
| **Belief that vaccine helped to prevent COVID-19 disease**, N^h^ (%) | | | | |
| Not at all | 44 (15.2) | 20 (45.5) | 24 (54.5) | 0.355 |
| Little | 61 (21.1) | 28 (45.9) | 33 (54.1) |  |
| Moderate | 77 (26.6) | 36 (46.8) | 41 (53.2) |  |
| A lot | 73 (25.3) | 25 (34.3) | 48 (65.7) |  |
| Very much | 34 (11.8) | 11 (32.4) | 23 (67.6) |  |
| **If you have been infected with COVID-19, how much do you think the vaccine reduce the symptoms of the disease**, N^i^ (%) | | | | |
| Not at all | 127 (17.4) | 68 (53.5) | 59 (46.5) | **0.012** |
| Little | 164 (22.4) | 80 (48.8) | 84 (51.2) |  |
| Moderate | 169 (23.1) | 70 (41.4) | 99 (58.6) |  |
| A lot | 200 (27.3) | 71 (35.5) | 129 (64.5) |  |
| Very much | 72 (9.8) | 29 (40.3) | 43 (59.7) |  |
| **If you have not received the COVID-19 vaccine to date, do you plan to receive it**, N^j^ (%) | | | | |
| No/I am not sure | 172 (96.1) | 99 (57.6) | 73 (42.4) | 0.441 |
| Yes | 7 (3.9) | 3 (42.9) | 4 (57.1) |  |
| ^a^N=1075; ^b^N=1073; ^c^N=1057; ^d^N=1062; ^e^N=925; ^f^N=924; ^g^N=938; ^h^N=289; ^i^N=732; ^j^N=179; ^k^Differences were tested using chi^2^ test; Bold values indicate statistically significant association at 5% significance level. | | | | |

**Supplementary Table 4.** Health status overall, and by fear of future epidemics and/or pandemics.

|  | **Overall**  (N=1075) | **Fear of future epidemics or pandemics** | | |
| --- | --- | --- | --- | --- |
|  |  | **No**  (N=498) | **Yes**  (N=577) | **p-value** |
| **Mobility**, N^a^ (%) | | | | |
| I have no problems in walking about | 974 (90.8) | 462 (47.4) | 512 (52.6) | **0.048**^e^ |
| I have some problems in walking about | 98 (9.1) | 35 (35.7) | 63 (64.3) |  |
| I am confined to bed | 1 (0.1) | 1 (100.0) | 0 (0.0) |  |
| **Self-care**, N^b^ (%) | | | | |
| I have no problems with self-care | 1055 (98.4) | 491 (46.5) | 564 (53.5) | 0.267^e^ |
| I have some problems washing or dressing myself | 16 (1.5) | 5 (31.2) | 11 (68.8) |  |
| I am unable to wash or dress myself | 1 (0.1) | 1 (100.0) | 0 (0.0) |  |
| **Usual activities**, N^a^ (%) | | | | |
| I have no problems with performing my usual activities | 1001 (93.3) | 473 (47.3) | 528 (52.7) | 0.109^e^ |
| I have some problems with performing my usual activities | 70 (6.5) | 24 (34.3) | 46 (65.7) |  |
| I am unable to perform my usual activities | 2 (0.2) | 1 (50.0) | 1 (50.0) |  |
| **Pain/Discomfort**, N^c^ (%) | | | | |
| I have no pain or discomfort | 830 (77.7) | 409 (49.3) | 421 (50.7) | **0.002**^e^ |
| I have moderate pain or discomfort | 230 (21.5) | 87 (37.8) | 143 (62.2) |  |
| I have extreme pain or discomfort | 8 (0.8) | 1 (12.5) | 7 (87.5) |  |
| **Anxiety/Depression**, N^d^ (%) | | | | |
| I am not anxious or depressed | 465 (44.0) | 254 (54.6) | 211 (45.4) | **<0.001**^e^ |
| I am moderately anxious or depressed | 526 (49.8) | 218 (41.4) | 308 (58.6) |  |
| I am extremely anxious or depressed | 66 (6.2) | 19 (28.8) | 47 (71.2) |  |
| **Mean score of visual analogue scale** ± SD | 70.2 ± 43.9 | 76.1 ± 54.0 | 65.1 ± 31.8 | **<0.001**^f^ |
| Abbreviations: SD; Standard Deviation; ^a^N=1073; ^b^N=1072; ^c^N=1068; ^d^N=1057; ^e^Differences were tested using chi^2^ test; ^f^Differences were tested using t-test; Bold values indicate statistically significant association at 5% significance level. | | | | |

**Supplementary Table 5.** Health literacy overall and by fear of future epidemics and/or pandemics.

|  | **Overall** (N=1075) | **Fear of future epidemics or pandemics** | | |
| --- | --- | --- | --- | --- |
|  |  | **No** (N=498) | **Yes** (N=577) | **p-value** |
| **Mean score*** (0-16) ± SD | 7.9 ± 5.9 | 6.8 ± 5.8 | 8.9 ± 5.7 | **<0.001**^a^ |
| **Health literacy** | | | | |
| Inadequate (0-8) | 556 (51.7) | 299 (53.8) | 257 (46.2) | **<0.001**^b^ |
| Problematic (9-12) | 191 (17.8) | 85 (44.5) | 106 (55.5) |  |
| Adequate (13-16) | 328 (30.5) | 114 (34.8) | 214 (65.2) |  |
| Abbreviations: SD; Standard Deviation; ^a^Differences were tested using t-test; ^b^Differences were tested using chi^2^ test; Bold values indicate statistically significant association at 5% significance level. Maximum score indicates worst health literacy. | | | | |

**Supplementary Table 6.** Trust in existing and future vaccine, in public health, science and medicine overall and by fear of future epidemics and/or pandemics.

|  | **Overall** (N=1075) | **Fear of future epidemics or pandemics** | | |
| --- | --- | --- | --- | --- |
|  |  | **No** (N=498) | **Yes** (N=577) | **p-value** |
| **Mean score*** (10-50) ± SD | 28.4 ± 7.6 | 27.6 ± 7.5 | 29.1 ± 7.7 | **0.0015**^e^ |
| **Level of trust**, N^a^ (%) | | | | |
| Low (≤23) | 300 (27.9) | 165 (55.0) | 135 (45.0) | **0.001**^d^ |
| Moderate (24-28) | 252 (23.4) | 103 (40.9) | 149 (59.1) |  |
| High (≥29) | 523 (48.7) | 230 (44.0) | 293 (56.0) |  |
| **To what extent do you trust licensed (approved) vaccines (not including COVID-19 vaccines)**, N^b^ (%) | | | | |
| No trust | 65 (6.1) | 40 (61.5) | 25 (38.5) | 0.154^d^ |
| Some trust | 187 (17.4) | 82 (43.8) | 105 (56.2) |  |
| Sufficient trust | 301 (28.0) | 136 (45.2) | 165 (54.8) |  |
| A lot of trust | 315 (29.3) | 145 (46.0) | 170 (54.0) |  |
| Great trust | 206 (19.2) | 95 (46.1) | 111 (53.9) |  |
| **To what extent do you trust the licensed (approved) vaccines against COVID-19**, N^a^ (%) | | | | |
| No trust | 186 (17.3) | 111 (59.7) | 75 (40.3) | **0.002**^d^ |
| Some trust | 286 (26.6) | 121 (42.3) | 165 (57.7) |  |
| Sufficient trust | 292 (27.2) | 135 (46.2) | 157 (53.8) |  |
| A lot of trust | 212 (19.7) | 88 (41.5) | 124 (58.5) |  |
| Great trust | 99 (9.2) | 43 (43.4) | 56 (56.6) |  |
| **How much would you trust new vaccines that might be developed (not just for COVID-19 vaccines)**, N^b^ (%) | | | | |
| No trust | 130 (12.1) | 66 (50.8) | 64 (49.2) | 0.541^d^ |
| Some trust | 331 (30.8) | 161 (48.6) | 170 (51.4) |  |
| Sufficient trust | 313 (29.1) | 141 (45.1) | 172 (54.9) |  |
| A lot of trust | 201 (18.7) | 86 (42.8) | 115 (57.2) |  |
| Great trust | 99 (9.3) | 44 (44.4) | 55 (55.6) |  |
| **To what extent do you trust the government and government agencies of the Republic of Cyprus regarding health issues**, N^b^ (%) | | | | |
| No trust | 244 (22.7) | 134 (54.9) | 110 (45.1) | **<0.001**^d^ |
| Some trust | 371 (34.5) | 179 (48.2) | 192 (51.8) |  |
| Sufficient trust | 297 (27.7) | 124 (41.8) | 173 (58.2) |  |
| A lot of trust | 125 (11.6) | 55 (44.0) | 70 (56.0) |  |
| Great trust | 37 (3.5) | 6 (16.2) | 31 (83.8) |  |
| **To what extent do you trust the public health authorities and the health care system of the Republic of Cyprus**, N^c^ (%) | | | | |
| No trust | 174 (16.2) | 96 (55.2) | 78 (44.8) | **0.005**^d^ |
| Some trust | 392 (36.5) | 186 (47.5) | 206 (52.5) |  |
| Sufficient trust | 312 (29.1) | 141 (45.2) | 171 (54.8) |  |
| A lot of trust | 151 (14.1) | 64 (42.4) | 87 (57.6) |  |
| Great trust | 44 (4.1) | 11 (25.0) | 33 (75.0) |  |
| **To what extent do you trust science in general,** N^c^ (%) | | | | |
| No trust | 10 (0.9) | 6 (60.0) | 4 (40.0) | 0.565^d^ |
| Some trust | 90 (8.4) | 46 (51.1) | 44 (48.9) |  |
| Sufficient trust | 292 (27.2) | 130 (44.5) | 165 (55.5) |  |
| A lot of trust | 374 (34.9) | 180 (48.1) | 194 (51.9) |  |
| Great trust | 307 (28.6) | 136 (44.3) | 171 (55.7) |  |
| **How much do you trust the medical community,** N^b^ (%) | | | | |
| No trust | 21 (2.0) | 13 (61.9) | 8 (38.1) | 0.178^d^ |
| Some trust | 145 (13.5) | 77 (53.1) | 68 (46.9) |  |
| Sufficient trust | 354 (32.9) | 157 (44.4) | 197 (55.6) |  |
| A lot of trust | 372 (34.6) | 173 (46.5) | 199 (53.5) |  |
| Great trust | 183 (17.0) | 78 (42.6) | 105 (57.4) |  |
| **To what extent do you trust health professionals (not including the medical community)**, N^a^ (%) | | | | |
| No trust | 26 (2.4) | 16 (61.5) | 10 (38.5) | **0.004**^d^ |
| Some trust | 177 (16.5) | 102 (57.6) | 75 (42.4) |  |
| Sufficient trust | 396 (36.9) | 172 (43.4) | 224 (56.6) |  |
| A lot of trust | 333 (31.0) | 150 (45.1) | 183 (54.9) |  |
| Great trust | 142 (13.2) | 57 (40.1) | 85 (59.9) |  |
| **To what extent do you trust the media on public health issues**, N^a^ (%) | | | | |
| No trust | 365 (34.0) | 192 (52.6) | 173 (47.4) | **<0.001**^d^ |
| Some trust | 477 (44.4) | 224 (47.0) | 253 (53.0) |  |
| Sufficient trust | 174 (16.2) | 66 (37.9) | 108 (62.1) |  |
| A lot of trust | 46 (4.3) | 15 (32.6) | 31 (67.4) |  |
| Great trust | 12 (1.1) | 1 (8.3) | 11 (91.7) |  |
| Abbreviations: SD; Standard Deviation; ^a^N=1075; ^b^N=1074; ^c^N=1073; ^d^Differences were tested using chi2 test; ^e^Differences were tested using t-test; Bold values indicate statistically significant association at 5% significance level. Maximum score indicates greater trust. | | | | |
